# Supplementary material for: Hydrogel-in-hydrogel live bioprinting for guidance and control of organoids and organotypic cultures
Source: Nat Commun. 2023 May 30;14:3128. doi: 10.1038/s41467-023-37953-4 (PMC10229611; doi:10.1038/s41467-023-37953-4)
Supplement: Supplementary file 1 — Supplementary Information [file 41467_2023_37953_MOESM1_ESM.pdf]

Supplementary Information for:

Hydrogel-in-hydrogel live bioprinting for guidance and control of organoids and organotypic cultures

Anna Urciuolo[1,2,¶,*], Giovanni Giuseppe Giobbe[3,¶], Yixiao Dong[4¶], Federica Michielin[3], Luca Brandolino[5,6], Michael Magnussen[3], Onelia Gagliano[5,6], Giulia Selmin[3], Valentina Scattolini[2], Paolo Raffa[2], Paola Caccin[7], Soichi Shibuya[3], Dominic Scaglioni[3], Xuechun Wang[4], Ju Qu[4], Marko Nikolic[3], Marco Montagner[1], Gabriel L. Galea[3], Hans Clevers[8], Monica Giomo[5], Paolo De Coppi[3,9], Nicola Elvassore[3,5,6*].

[1] Dept. of Molecular Medicine, University of Padova, Padova, Italy

[2] Istituto di Ricerca Pediatrica, Città della Speranza, Padova, Italy

[3] GOSICH Zayed Centre for Research into Rare Disease in Children, University College London, London, UK

[4] Shanghai Institute for Advanced Immunochemical Studies (SIAIS), ShanghaiTech University, Shanghai, China

[5] Dept. of Industrial Engineering, University of Padova, Padova, Italy

[6] Veneto Institute of Molecular Medicine, Padova, Italy

[7] Dept. of Biomedical Science, University of Padova, Padova, Italy

[8] Hubrecht Institute, KNAW and University Medical Center, Utrecht, The Netherlands

[9] Dept. of Specialist Neonatal and Paediatric Surgery, Great Ormond Street Hospital, London, UK

¶ These authors contributed equally: Anna Urciuolo, Giovanni Giuseppe Giobbe & Yixiao Dong

* Co-corresponding authors:

Nicola Elvassore, nicola.elvassore@unipd.it; Anna Urciuolo, anna.urciuolo@unipd.it

This file contains:

- Supplementary Figures 1-7

- Supplementary Tables 1-6

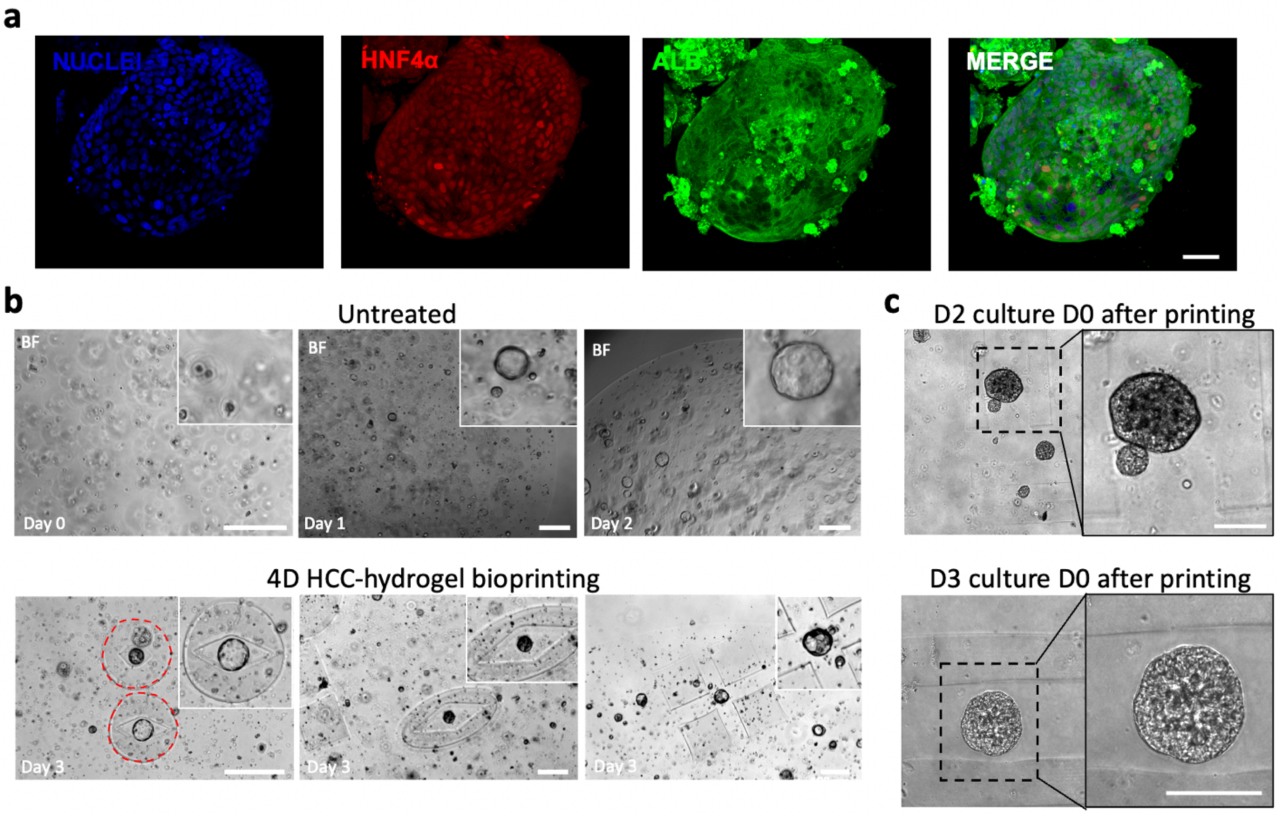

Supplementary Figure 1.

Characterization of human fetal hepatocyte organoids. a) Immunofluorescence panel showing characterization of human fetal hepatocyte organoids derived from post-conception week (PCW) 8 fetus. Nuclei shown in blue, nuclear hepatocyte nuclear factor 4 alpha (HNF4α) shown in red, cytoplasmic human albumin (ALB) shown in green. Scale bar 30 μm. **b)** Representative bright field images of single cell-derived human liver organoids after 1, 2 or 3 days of culture in untreated conditions (upper panels) or after live bioprinting at day 3 of human liver organoid culture (bottom panels). HCC-Gelatin hydrogels were fabricated with the desired shape to accommodate the selected organoid grown in the 3D culture. Red dashed lines indicate the hydrogel margins. Scale bars, 100 μm. **c)** Representative bright field images showing cell clump-derived human liver organoids after 2 (top panel) or 3 (lower panel) days of culture and live bioprinting of parallelepiped shaped HCC-Gelatin hydrogels. Inserts show higher magnification. Red dashed lines indicate the hydrogel margins. Scale bars, 100 μm.

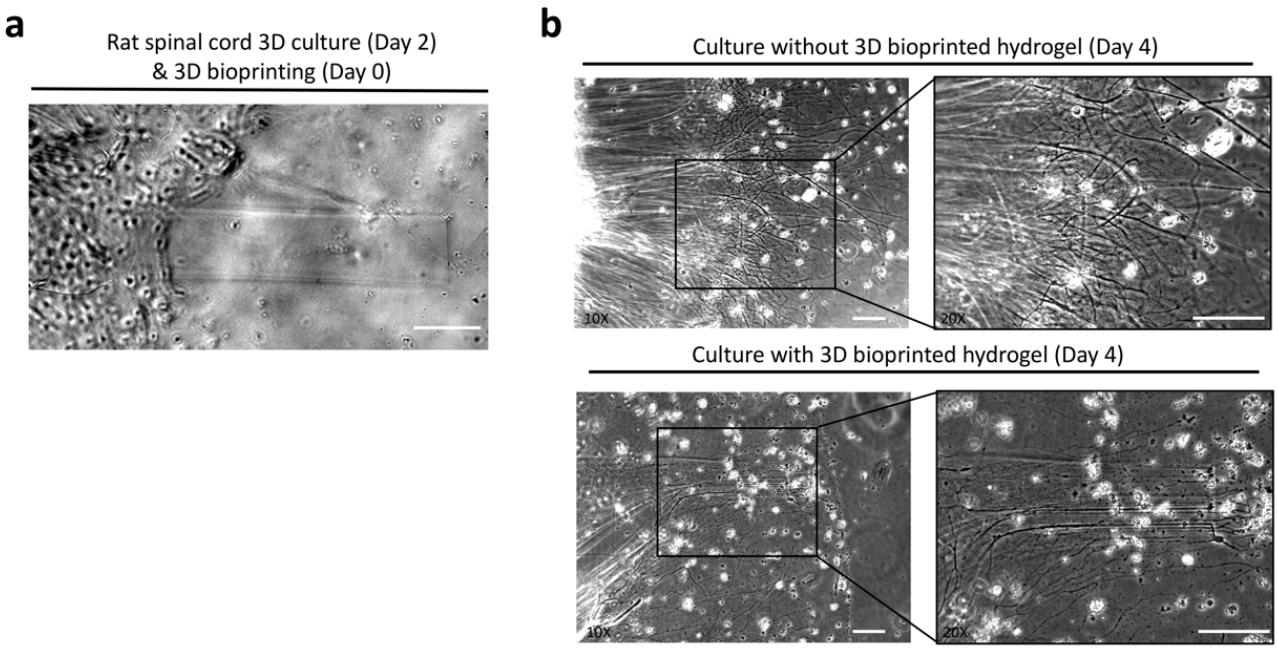

Supplementary Figure 2.

Hydrogel-in-hydrogel live bioprinting for neural axon guidance. a) Phase contrast imaging shows the presence of hydrogel structures after printing which are precisely fabricated according to the initial design. Scale bar, 50 µm. **b)** Phase contrast imaging performed 2 days after 3D bioprinting (4 days of spinal cord culture) showed that axons localized close to the fabricated hydrogel could sprout over the hydrogel and were disposed in an organized pattern according to the printed shape, while in absence of a hydrogel structure, axons were randomly oriented. Scale bar, 50 µm.

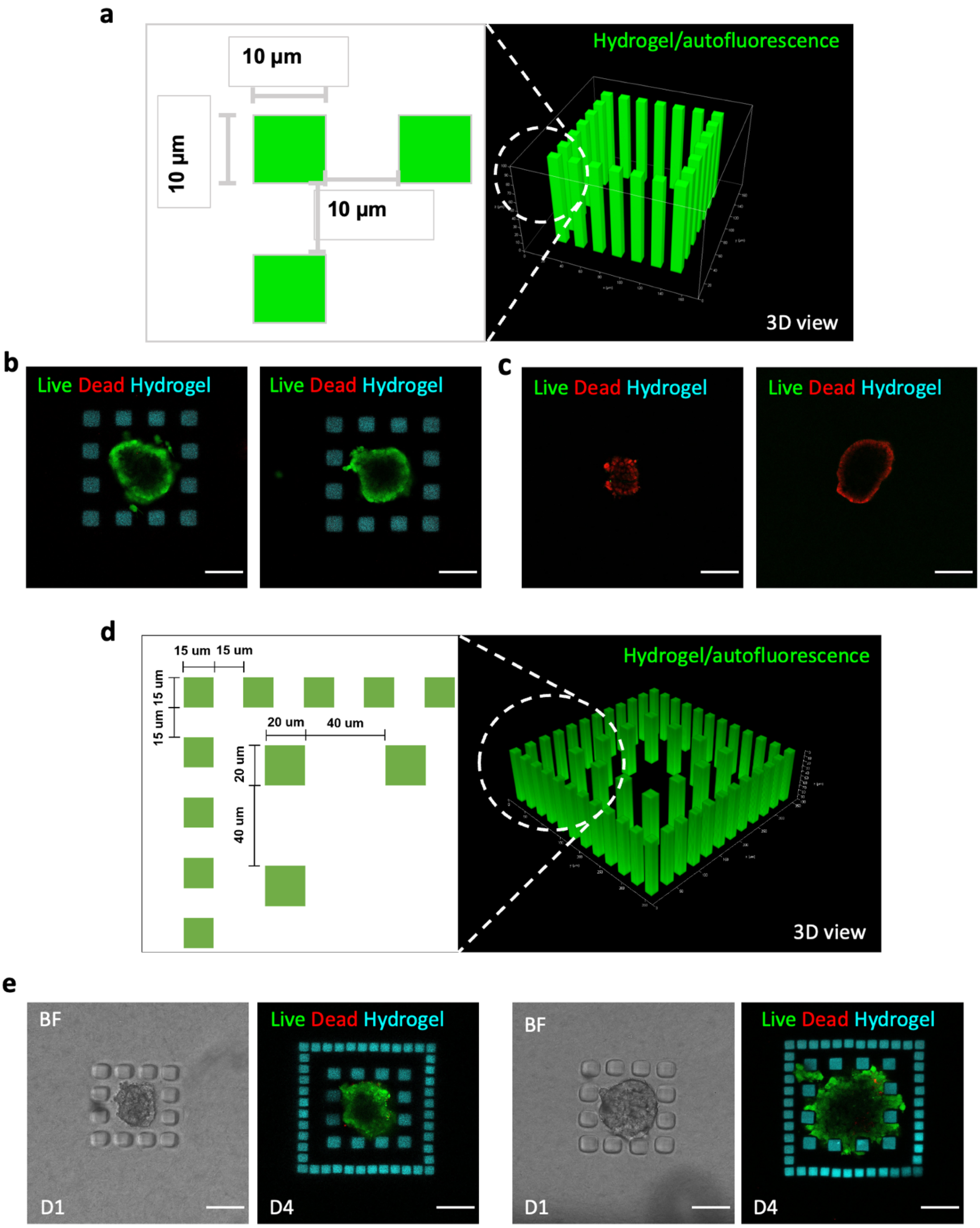

Supplementary Figure 3.

Hydrogel-in-hydrogel design and printing for cancer organoids. a) Left panel, schematic view of the pillar (green) design. Right panel, 3D reconstruction of PEG-based pillars (green) printed within the gelatin droplet. **b)** Representative fluorescence images showing live (green) and dead (red) staining of two independent experiments of 3D cancer organoid cultures growth for 1 day before hydrogel-in-hydrogel bioprinting and

analyzed one day after bioprinting. Hydrogels are shown in light blue. Scale bars, 100 µm. **c)** Representative fluorescence images showing live (green) and dead (red) staining of two independent experiments of 3D cancer organoid culture growth for 2 days and treated with MeOH to induce cell dead. Scale bars, 100 µm. **d)** Left panel, schematic view of the pillar (green) design for additive printing at multiple time points. Right panel, 3D reconstruction of PEG-based pillars (green) printed within the gelatin droplet. **e)** Representative fluorescence images showing two independent experiments of 3D cancer organoid culture growth for 1 day before the first hydrogel-in-hydrogel bioprinting (BF) and subjected to the second bioprinting step 2 days after culture (fluorescence images). Live (green) and dead (red) staining was analyzed one day after the second bioprinting (for a total 4 days in culture). Hydrogels are shown in light blue. Scale bars, 100 µm.

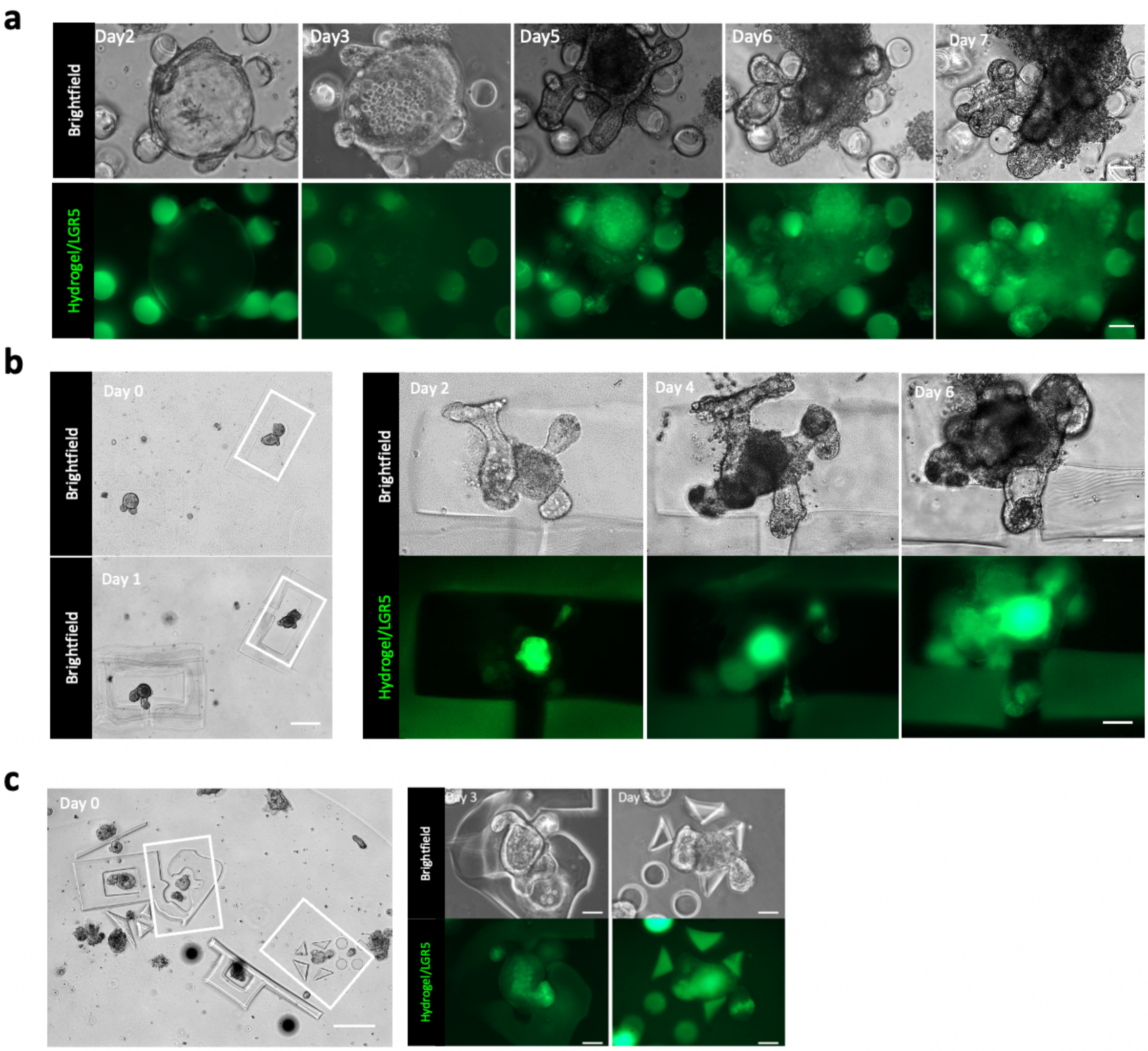

Supplementary Figure 4.

Hydrogel-in-hydrogel bioprinting integrated in murine LGR5-EGFP SIO 3D cultures. a) Representative bright field (upper) and fluorescence (lower) images of SIOs after 2, 3, 5, 6 or 7 days of culture and hydrogel-in-hydrogel bioprinting. HCC-gel hydrogels were fabricated as pillars in proximity of the interested organoid to follow SIO budding during the time of culture. Scale bars, 50 µm. **b)** Left, representative bright field images of SIOs at the moment (day 0) and 1 day after culture and hydrogel-in-hydrogel bioprinting. Right, representative bright field (upper) and fluorescence (lower) images of murine SIOs after 2, 4 or 6 days of culture and hydrogel-in-hydrogel bioprinting with HCC-4-arm PEG hydrogels. Hydrogels were fabricated as rectangular walls with a single aperture in proximity of the interested organoids to force single events of SIO budding from the aperture during the time of culture. Scale bars, 50 µm. **c)** Left, representative bright field images of SIOs just after 4D bioprinting (day 0). Scale bar 100 µm. Right, representative bright field (upper) and fluorescence (lower) images of SIOs after 3 days of culture and hydrogel-in-hydrogel bioprinting of HCC-gel hydrogels. Hydrogels were fabricated with a desired shape to accommodate the selected organoid or as pillars in the same SIO culture via multiple ROI setup. Scale bars, 50 µm.

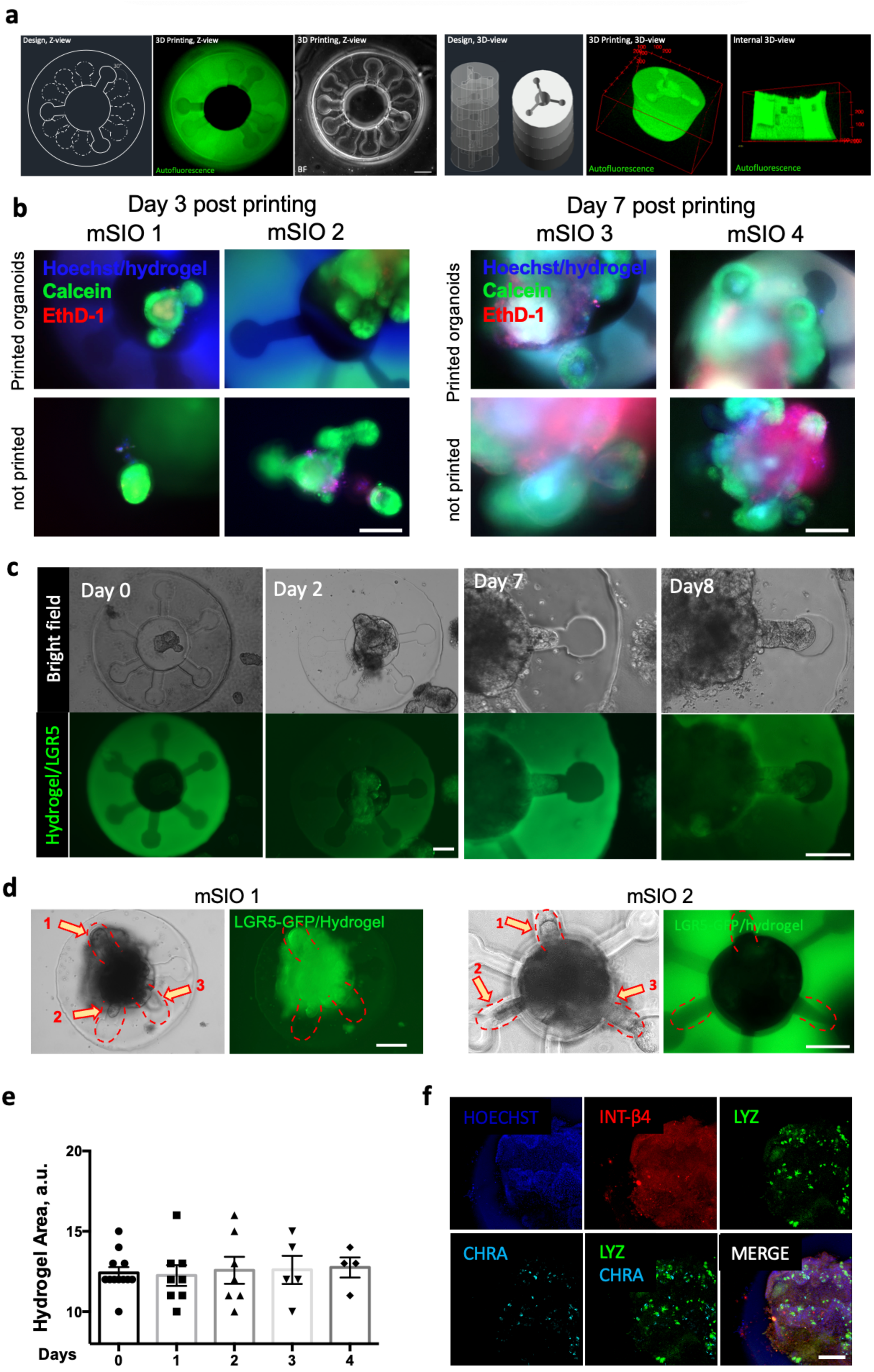
a
Design, Z-view
3D Printing, Z-view
Autofluorescence
3D Printing, Z-view
BF
Design, 3D-view
3D Printing, 3D-view
Autofluorescence
Internal 3D-view
Autofluorescence
b
Day 3 post printing
mSIO 1
mSIO 2
Day 7 post printing
mSIO 3
mSIO 4
Printed organoids
not printed
Hoechst/hydrogel
Calcein
EthD-1
c
Bright field
Hydrogel/LGR5
Day 0
Day 2
Day 7
Day8
d
mSIO 1
mSIO 2
LGR5-GFP/Hydrogel
1
2
3
e
Hydrogel Area, a.u.
20
15
10
Days
0
1
2
3
4
f
HOECHST
INT-β4
LYZ
CHRA
LYZ
CHRA
MERGE

Supplementary Figure 5.

Hydrogel-in-hydrogel bioprinting integrated in murine LGR5-EGFP SIO 3D cultures at supra-organoid level. a) Representative bright field and fluorescence images showing primordial 30° small intestine design and HCC-gel hydrogels (left panel, top view; right panel, 3D reconstruction view). Scale bar 100 µm. **b)** Live/Dead staining showing calcein (green) live cells and ethidium homodimer-1 (red) dead cells in mSIOs printed and unprinted after 3 and 7 days of culture, in 4 different samples. Scale bars 100 µm. **c)** Representative bright field (upper) and fluorescence (lower) images of mSIOs just after primordial small intestine HCC-gel hydrogel printing or 2, 7 or 8 days of culture and bioprinting. Budding was observed according to the defied shape of the hydrogel. Scale bar 100 µm. **d**) Representative bright field and fluorescence images showing mSIO buds invading multiple crypts at different Z-levels of the primordial small intestine design after 6 days of culture post-printing. mSIO 1 panoramic view of main Fig. 4c. Scale bar 100 µm for mSIO 1, 200 µm for mSIO 2. **e)** Quantification of the hydrogel area during culture. Data are shown as mean ± s.d. of 3 independent replicates. No statistically significant differences were observed after multiple comparison one-way ANOVA analysis. **f)** Representative images showing immunofluorescence analysis for INT-β4 (red), LYZ (green) and CHRA (lightblue) of mSIO cultured for 10 days within the primordial small intestine-shaped HCC-gel hydrogel. Nuclei are stained with Hoechst (blue). Scale bar 100 µm.

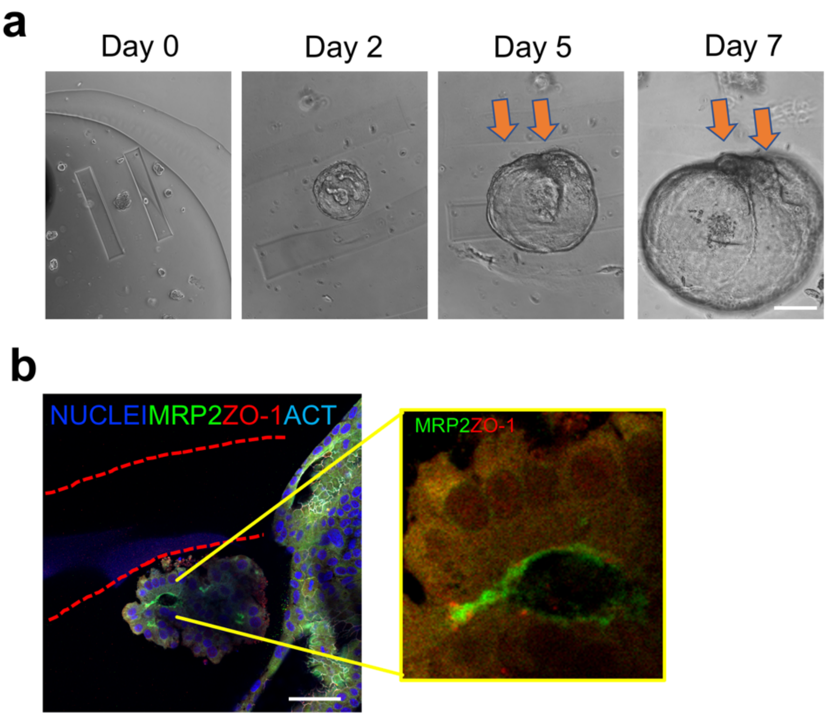

Supplementary Figure 6.

Hydrogel-in-hydrogel bioprinting for human liver organoid polarization. a) Bright field images showing adjacent walls not touching the growing hepatocyte organoid after 7 days of culture. Scale bar 100 μm. **b)** Immunofluorescent panels showing polarized organoid in correspondence with the printed adjacent walls (red dotted lines). Nuclei shown in blue, multidrug resistance-associated protein 2 (MRP2) shown in green, zonula occludens-1 (ZO-1) shown in red, f-actin (ACT) shown in cyan. Scale bar 50 μm.

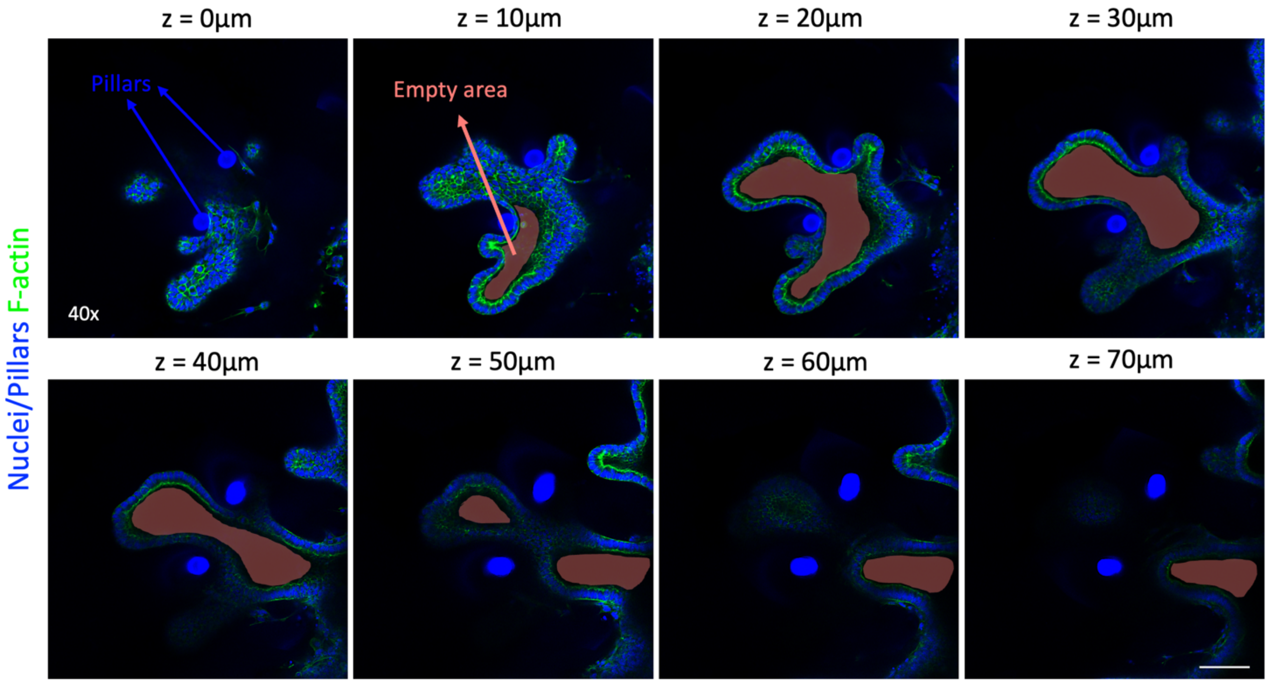

Supplementary Figure 7.

Hydrogel-in-hydrogel bioprinting for human liver organoid polarization. Immunofluorescent panel showing lung tip branching in between two pillars (blue) and inner (luminal) polarity maintained (F-actin, green). The hollow segments were highlight in dark pink (empty area). Nuclei are stained with Hoechst (blue). Scaler bar 100 µm.

Supplementary Table 1: Statistical significance of Fig. 4d

Tukey's multiple comparisons test	Mean Diff,	95% CI of diff,	Significant?	Summary
1 vs. 2	-0,7983	-4,945 to 3,348	No	ns
1 vs. 3	-2,574	-6,720 to 1,573	No	ns
1 vs. 4	-4,323	-8,469 to -0,1760	Yes	*
1 vs. 5	-7,811	-12,48 to -3,140	Yes	***
1 vs. 6	-12,32	-16,99 to -7,644	Yes	****
2 vs. 3	-1,775	-6,079 to 2,528	No	ns
2 vs. 4	-3,524	-7,827 to 0,7788	No	ns
2 vs. 5	-7,013	-11,82 to -2,202	Yes	**
2 vs. 6	-11,52	-16,33 to -6,706	Yes	****
3 vs. 4	-1,749	-6,052 to 2,554	No	ns
3 vs. 5	-5,237	-10,05 to -0,4263	Yes	*
3 vs. 6	-9,741	-14,55 to -4,930	Yes	****
4 vs. 5	-3,488	-8,300 to 1,323	No	ns
4 vs. 6	-7,993	-12,80 to -3,182	Yes	***
5 vs. 6	-4,504	-9,774 to 0,7661	No	ns

Supplementary Table 2: Statistical significance of Fig. 4e.

Tukey's multiple comparisons test	Mean Diff,	95% CI of diff,	Significant?	Summary
1 vs. 2	-0,764	-3,171 to 1,643	No	ns
1 vs. 3	-2,043	-4,643 to 0,5570	No	ns
1 vs. 4	-5,398	-8,346 to -2,449	Yes	***
1 vs. 5	-5,754	-9,014 to -2,495	Yes	***
2 vs. 3	-1,279	-3,879 to 1,321	No	ns
2 vs. 4	-4,633	-7,582 to -1,685	Yes	***
2 vs. 5	-4,99	-8,250 to -1,731	Yes	**
3 vs. 4	-3,354	-6,462 to -0,2469	Yes	*
3 vs. 5	-3,711	-7,116 to -0,3071	Yes	*
4 vs. 5	-0,3568	-4,034 to 3,320	No	ns

Supplementary Table 3: Mouse small intestinal organoid medium

Component	Stock conc.	Final conc.
Advanced DMEM F-12 (Thermo 12634)	-	To volume
HEPES (Thermo 15630080)	1 M	10 mM
Glutamax (Thermo 35050061)	100 X	2 mM
B-27 supplement minus vitamin A (Thermo 12587010)	50 X	1 X
n-acetylcysteine (Sigma A9165)	500 mM	1.25 mM
Pen/Strep (Thermo 15140122)	100 %	1 %
Wnt-3A (Peprotech 315-20) optional	50 µg/mL	100 ng/mL
R-spondin 1 (Peprotech 120-38)	100 µg/mL	500 ng/mL
Noggin (R&D 6057-NG)	100 µg/mL	100 ng/mL
EGF (Thermo PMG8043)	500 µg/mL	50 ng/mL

Supplementary Table4: Human fetal hepatocyte organoid medium

**Component**	**Stock conc.**	**Final conc.**
Advanced DMEM F-12 (Thermo 12634028)	-	To volume
Penicillin Streptomycin (Thermo 15140122)	100%	1%
Glutamax (Thermo 10378016)	100 X	1X
HEPES (Thermo 15630080)	1 M	10 mM
B-27 supplement (Gibco 17504-044)	50 X	1 X
n-acetylcysteine (Sigma A9165)	500 mM	1.25 mM
R-spondin (Peprotech 120-38)	100 µg/mL	100 ng/mL
EGF (Thermo PMG8043)	500 µg/mL	50 ng/mL
Gastrin (Sigma G9020)	100 µM	10 nM
Nicotinamide (Sigma 72340)	1 M	10 mM
FGF7 (Peprotech 100-19)	50 µg/mL	100 ng/mL
FGF10 (Peprotech 100-26)	100 µg/mL	100 ng/mL
HGF (Peprotech 100-39)	20 µg/mL	50 ng/mL
GSK-3 inhibitor (CHIR 99021) (Tocris 4423)	3 mM	3 µM
TGFβi (A83-01) (Tocris 2939)	5 mM	2 µM
TGFα (Peprotech 100-16A)	20 µg/mL	20 ng/mL
Primocin (Thermo)	50mg/mL	100 µg/mL
For differentiation: Oncostatin M (Peprotech)	20 µg/mL	10 ng/mL
For differentiation Dexamethasone	1 mM	1 µM

Supplementary Table 5: Mouse fetal lung organoid medium

Component	Stock conc.	Final conc.
Advanced DMEM F-12 (Thermo 12634028)	-	To volume
Penicillin Streptomycin (Thermo 15140122)	100%	1%
Glutamax (Thermo 10378016)	100 X	1X
HEPES (Thermo 15630080)	1 M	10 mM
Heparin solution (Sigma-Aldrich)	100%	1%
EGF (Thermo PMG8043)	500 µg/mL	50 ng/mL
FGF9 (Peprotech 100-23)	50 µg/mL	50 ng/mL
FGF10 (Peprotech 100-26)	100 µg/mL	50 ng/mL
GSK-3 inhibitor (CHIR 99021) (Tocris 4423)	3 mM	3 µM
TGFβi (A83-01) (Tocris 2939)	5 mM	1 µM
Insulin-Transferrin-Selenium ITS (Thermo)	100%	1%
p38- MAPK pathway inhibitor BIRB-796	1 mM	1 µM
ROCK inhibitor Y-27632 (Tocris 1254)	10 mg/mL	10 µg/mL

Supplementary Table 6: Antibody and molecule list

**Antibody/conjugated molecule**	**Dilution**
Ezrin (Thermo PA5-29358)	1:100
rFABP1 (R&D AF1565)	1:100
E-cadherin (BD 610182)	1:100
Olfactomedin-4 (Cell signaling 14369S)	1:50
Zonula occludens-1 (Invitrogen 40-2200)	1:200
MRP2 (Abcam ab3373)	1:100
Sox9 (R&D/AF3075)	1:100
Phalloidin 488 (Thermo A12379)	1:200
Goat anti-Rabbit 594 (Thermo A11012)	1:500
Goat anti-Rabbit 568 (Thermo A11011)	1:500
Goat anti-Rabbit 488 (Thermo A11008)	1:500
Goat anti-Mouse 488 (Thermo A11001)	1:500
Goat anti-Mouse 568 (Thermo A10037)	1:500
Donkey anti-Goat 647 (Thermo A-21447)	1:500
Hoechst 33342 (Thermo H1399)	10 µg/mL
