# Supplementary material for: Hydrogel-in-hydrogel live bioprinting for guidance and control of organoids and organotypic cultures
Source: Nat Commun. 2023 May 30;14:3128. doi: 10.1038/s41467-023-37953-4 (PMC10229611; doi:10.1038/s41467-023-37953-4)
Supplement: Supplementary file 3 — Description of additional supplementary files [file 41467_2023_37953_MOESM3_ESM.pdf]

Description of additional supplementary files

Supplementary Video 1.

Response of cancer organoids to PEG-based pillars fabricated via hydrogel-in-hydrogel bioprinting. Representative bright field time-lapse imaging of cancer organoid culture in presence or absence (right panel) of CMMC-PEG hydrogels pillars fabricated around a hydrogel-embedded growing tumor organoid (seeded 1 day before bioprinting).

Supplementary Video 2.

Supra-organoid hydrogel-in-hydrogel bioprinting. Representative images showing a 3D reconstruction of the HCC-Gelatin primordial intestine-shaped hydrogel. The entire (right) or the cropped internal view (left panel) of the hydrogel is shown.

Supplementary Video 3.

Response of organotypic lung culture to PEG-based pillars fabricated via hydrogel-in-hydrogel bioprinting. Representative bright field time-lapse imaging of organotypic lung culture in presence of HCC-PEG hydrogels pillars.

Supplementary Video 4.

Apical constriction of F-actin in organotypic lung culture after pillar-induced bifurcation. Representative fluorescence Z-stack imaging of F-actin (green) and nuclei (blue, Hoechst) in organotypic lung culture in presence of HCC-PEG hydrogels pillars, showing cell apical sides facing luminal portion of the rudiments.

Supplementary Video 5.

Prolonged culture of lung rudiments after pillar induced bifurcation. Representative immunofluorescence Z-stack imaging of SOX9 (cyan), SOX2 (red) and nuclei (blue, Hoechst) in organotypic lung culture in presence of HCC-PEG hydrogels pillars. No fusion of different tips has been observed, even after a prolonged period of culture, i.e. 3 days.
